# Sauropod dinosaur teeth from the lower Upper Cretaceous Winton Formation of Queensland, Australia and the global record of early titanosauriforms

**DOI:** 10.1098/rsos.220381

**Published:** 2022-07-13

**Authors:** Stephen F. Poropat, Timothy G. Frauenfelder, Philip D. Mannion, Samantha L. Rigby, Adele H. Pentland, Trish Sloan, David A. Elliott

**Affiliations:** ^1^ Australian Age of Dinosaurs Natural History Museum, The Jump-Up, Winton, Queensland 4735, Australia; ^2^ School of Science, Computing and Engineering Technologies, Swinburne University of Technology, John Street, Hawthorn, Victoria 3122, Australia; ^3^ School of Environmental and Rural Science, University of New England, Armidale, New South Wales 2351, Australia; ^4^ Department of Earth Sciences, University College London, Gower Street, London WC1E 6BT, UK

**Keywords:** Winton Formation, Sauropoda, Diamantinasauria, Titanosauriformes, palaeobiogeography, microwear

## Abstract

The Upper Cretaceous Winton Formation of Queensland, Australia, has produced several partial sauropod skeletons, but cranial remains—including teeth—remain rare. Herein, we present the first description of sauropod teeth from this formation, based on specimens from three separate sites. An isolated tooth and a dentary fragment from the *Diamantinasaurus matildae* type locality are considered to be referable to that titanosaurian taxon. A single tooth from the *D. matildae* referred specimen site is similarly regarded as being part of that individual. Seventeen teeth from a new site that are morphologically uniform, and similar to the teeth from the two *Diamantinasaurus* sites, are assigned to Diamantinasauria. All sauropod teeth recovered from the Winton Formation to date are compressed-cone-chisel-shaped, have low slenderness index values (2.00–2.88), are lingually curved at their apices, mesiodistally convex on their lingual surfaces, and lack prominent carinae and denticles. They are markedly different from the chisel-like teeth of derived titanosaurs, more closely resembling the teeth of early branching members of the titanosauriform radiation. This provides further support for a ‘basal’ titanosaurian position for Diamantinasauria. Scanning electron microscope microwear analysis of the wear facets of several teeth reveals more scratches than pits, implying that diamantinasaurians were mid-height (1–10 m) feeders. With a view to assessing the spatio-temporal distribution of sauropod tooth morphotypes before and after deposition of the Winton Formation, we provide a comprehensive continent-by-continent review of the early titanosauriform global record (Early to early Late Cretaceous). This indicates that throughout the Early–early Late Cretaceous, sauropod faunas transitioned from being quite diverse at higher phylogenetic levels and encompassing a range of tooth morphologies at the start of the Berriasian, to faunas comprising solely titanosaurs with limited dental variability by the end-Turonian. Furthermore, this review highlights the different ways in which this transition unfolded on each continent, including the earliest records of titanosaurs with narrow-crowned teeth on each continent.

## 1. Introduction

Sauropod dinosaur teeth are exceptionally rare in Australia, despite being relatively commonly preserved elements in Jurassic–Cretaceous deposits elsewhere (e.g. [1–6]). This is perhaps especially surprising given the burgeoning sauropod fossil record from the lower Upper Cretaceous Winton Formation of Queensland, which includes cranial remains [7–21]. To date, the approximately stratigraphically equivalent (Cenomanian) Griman Creek Formation of Lightning Ridge, New South Wales, is the only sedimentary unit in Australia from which sauropod teeth have been described [11,22]. The first sauropod tooth from Australia was reported by Molnar [23,24], although there was some uncertainty over its precise provenance, since ‘it was purchased from a chap who could not remember where he had obtained it’ [25, p. 334]. The tooth was regarded as being broadly similar to those of *Giraffatitan brancai* [26], and tentatively assigned to Brachiosauridae by Molnar [23], an interpretation that was followed in later works (e.g. [27]). A cast of this tooth, accessioned in the Queensland Museum (QM F10230), was listed in the brief synopsis of Australian sauropod records that prefaced the first work on sauropod material from the Winton Formation [7]. In December 1984, two sauropod teeth were purchased by the Australian Museum as part of the ‘Galman Collection’: AM F66769 and AM F66770 (the tooth from which QM F10230 was cast). One of these (AM F66769) was illustrated the following year in a popular article [28], and subsequent references to sauropod teeth from Lightning Ridge were invariably in the plural (e.g. [29]), even though AM F66770 was regarded as only ‘probably’ from Lightning Ridge [11,30]. AM F66769 and AM F66770 were described in detail by Molnar & Salisbury [11], and both were assigned to Titanosauriformes. Photographs of these teeth, and two others, appeared in a popular book [31], an unpublished thesis [32], and a review of the Lightning Ridge fossil assemblage [33]. Recently, Frauenfelder *et al.* [22] described 25 sauropod teeth from Lightning Ridge, including AM F66769 and AM F66770. Five tooth morphotypes were recognized, but these were regarded as representing as few as two taxa: a non-titanosaurian titanosauriform and a non-lithostrotian titanosaur.

Until recently, the rich sauropod fossil record from the Winton Formation did not include any teeth [7–12,14–21]; however, although teeth have now been reported from a site hosted within this unit near Eromanga [13], these remain undescribed. In mid-2019, excavations by the Australian Age of Dinosaurs Museum of Natural History (AAOD) at the ‘Mitchell’ site (AODL 270) on Elderslie Station (west of Winton, Queensland) produced a dozen isolated sauropod teeth, in addition to scattered postcranial remains. This discovery prompted a thorough search of the AAOD collection for additional sauropod teeth from other sites, which proved fruitful: several fragmentary sauropod teeth and jaw elements had been collected but had either been overlooked or misidentified. Prominent among these are: a sauropod jaw fragment with teeth, as well as an isolated tooth, from AODL 85 (the ‘Matilda’ site, Elderslie Station), found in association with the type specimen of *Diamantinasaurus matildae* (AODF 603 [12,15]); and a partial sauropod tooth crown from AODL 127 (the ‘Alex’ site, Belmont Station), found some 10 m from the cranial remains of a referred specimen of *D. matildae* (AODF 836 [16,19]). A second excavation at the ‘Mitchell’ site in mid-2021 produced at least five additional sauropod teeth, bringing the total therefrom to 17.

Here, we provide the first description of sauropod teeth from the Winton Formation. We also analyse their microwear, providing an updated inference of the dietary palaeoecology of these Australian sauropods. Finally, we compare these teeth with those of other sauropods, providing an overview of the Early–mid-Cretaceous global record, with an emphasis on titanosauriforms.

*Institutional abbreviations:* AAOD, Australian Age of Dinosaurs Natural History Museum, Winton, Queensland, Australia; AM, Australian Museum, Sydney, Australia; AODF, Australian Age of Dinosaurs Fossil; AODL, Australian Age of Dinosaurs Locality; LRF, Australian Opal Centre, Lightning Ridge, New South Wales, Australia; QM, Queensland Museum (Brisbane, Australia).

## 2. Geological setting

**Figure 1.**
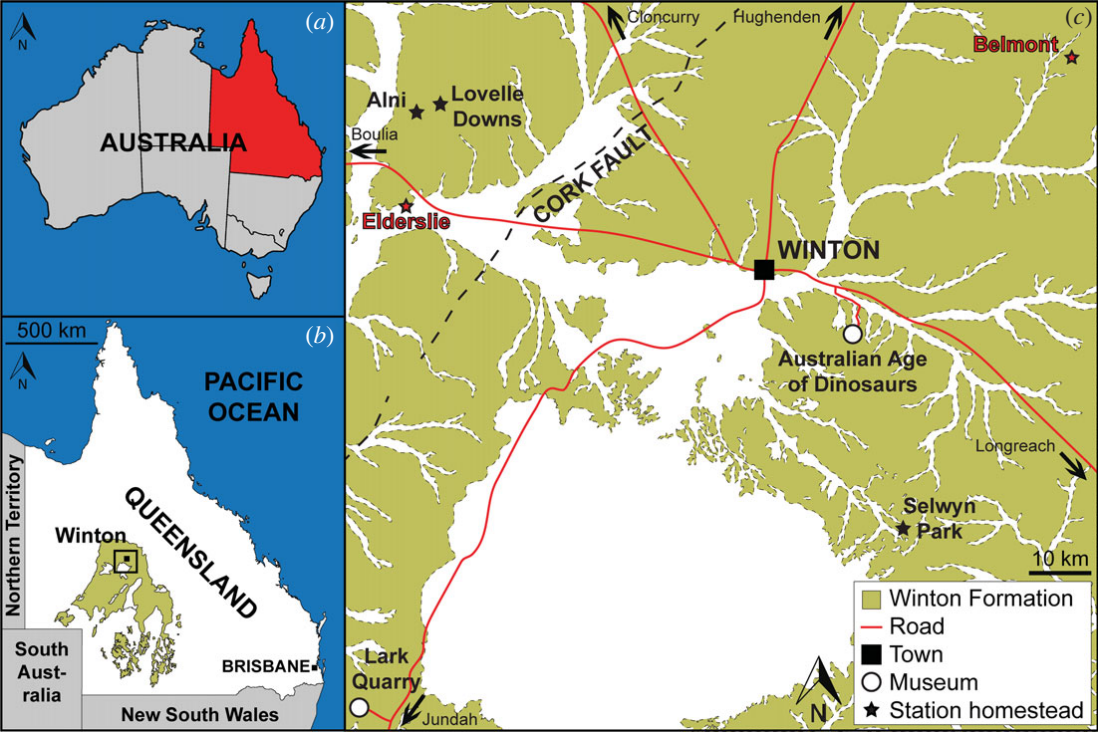
(*a*) Map of Australia with Queensland highlighted (modified from Poropat *et al*. [16]). (*b*) Map of Queensland with the distribution of Winton Formation outcrop plotted (modified from Poropat *et al*. [16]). (*c*) Map of Winton with the Winton Formation outcrop plotted, the location of Elderslie and Belmont Stations indicated, and numerous other cattle/sheep stations and sites in the region from which sauropod dinosaur remains have been collected and/or in which they are on display. Map drafted by the senior author (S.F.P.) in Adobe Illustrator CC 2017 (modified from Pentland *et al*. [34]), incorporating geological information from Vine [35] and Vine & Casey [36] (© Commonwealth of Australia (Geoscience Australia) 2021. This product is released under the Creative Commons Attribution 4.0 International Licence. http://creativecommons.org/licenses/by/4.0/legalcode).

The Winton Formation is a non-marine, mid-Cretaceous sedimentary unit that crops out extensively across the Eromanga Basin of northeast Australia (figure 1; [37–39]). It conformably overlies the marine Mackunda Formation and is weathered such that it has become clay-rich alluvium (colloquially termed ‘black soil’) across much of the Winton area [38]. The Winton Formation is dominated by volcanogenic sandstones and siltstones [40], which are presumed to be derived from the Whitsundays Volcanic Province to the east [41,42], and are thought to have accelerated (or even caused) the regression of the Eromanga Sea during the late Albian [43]. The Winton Formation spans the uppermost Albian to lowermost Turonian, with exposures near Winton thought to be predominantly Cenomanian in age [41,44]. The syndepositional palaeoclimate in northeast Australia was warm, seasonal and characterized by high rainfall; under this climatic regime, meandering rivers wound across the vast, low relief floodplain, with more permanent water bodies and heavily vegetated areas scattered across the landscape [45].

The geology of the ‘Matilda’ (AODL 85 (AODF 603 = *D. matildae* type specimen)) and ‘Alex’ (AODL 126 (AODF 836 = *D. matildae* referred specimen)) sites has been discussed elsewhere [12,15,19]: both were interpreted as abandoned channel systems, with the latter cross-cut by a minor subsequent channel. By contrast, the ‘Mitchell’ site is interpreted as a high-energy river channel deposit: it is characterized by a series of coarse siltstones and fine sandstones—with minor coal inclusions and rip-up sandstones, conglomerates and rounded claystone clasts—that overlies a fine, grey claystone from which it is separated by a sharp boundary.

Although it is plausible that the sauropod teeth at the ‘Mitchell’ site, as well as the numerous scattered sauropod bones, pertain to a single individual, we only tentatively infer this at this stage. This caution is warranted because several of the bones that were observed *in situ* show evidence of transportation prior to burial. Furthermore, the non-dental sauropod remains from the site have not yet been prepared, precluding assessment of element duplication or size-congruence. The high-energy nature of the palaeoenvironment, as well as the allochthonous nature of the assemblage, is highlighted by the fact that numerous non-sauropodan fossils are also present in the ‘Mitchell’ site, intermingled with the sauropod remains. These include: megaraptorid theropod teeth (similar to those of *Australovenator wintonensis* [12,46]); an anhanguerid pterosaur tooth (almost identical to some of those of *Ferrodraco lentoni* [34,47]; A.H. Pentland 2021, personal observation); numerous crocodyliform teeth, osteoderms and long bones; turtle fragments; several teeth that might pertain to plesiosaurs; a lungfish tooth plate referable to *Metaceratodus ellioti* [48]; unionid bivalves (including one referable to *Hyridella* (*Protohyridella*) *goondiwindiensis* [49]); conifer cones referable to *Austrosequoia wintonensis* [50] and *Emwadea microcarpa* [51]; and angiosperm leaves.

## 3. Methods

Several of the teeth involved in this study were surface scanned with an Artec Space Spider handheld laser scanner (www.artec3d.com/portable-3d-scanners/artec-spider-v2), which generates three-dimensional models using structured light. The three-dimensional models were manipulated and screenshot in Artec Studio 15 Professional (www.artec3d.com/3d-software/artec-studio).

Microwear analysis was conducted on five teeth from the ‘Mitchell’ site (AODF 963, AODF 984, AODF 985, AODF 1285 and AODF 1531). These teeth were selected because they preserved clear wear facets, and because they were found in 2019 (those found in 2021 were discovered too late for inclusion in these analyses). Each tooth was moulded using Pinkysil—a fast-set silicone—and cast using Easycast—a fast-set rigid polyurethane. Prior to moulding, each tooth was cleaned with acetone to ensure that no organic material was left on wear surfaces. Each cast was gold-coated using a NeoCoater MP-19020NCTR for a minimum of 2 min per wear surface. Once coated, teeth were examined under a JOEL-JSM-6010LA scanning electron microscope (SEM) at the University of New England, Armidale. Images were produced using InTouch Scope v. 1.10.

Identification and analysis of microwear features was implemented within RStudio v. 1.3.1093 using the R-package ‘MicroWeaR’ v. 1.1.0 [52,53]. The latter is a freely available software used to examine and score microwear features in a semi-automatic way [52]. Microwear features were classified as either large or small pits, and fine or coarse scratches. Classification of each feature is as follows: if the length/width ratio is less than or equal to 4 µm it is considered a pit, if greater than 4 µm it is considered a scratch [53]. If a pit has a diameter of less than or equal to 8 µm it is considered a small pit, while if a scratch has a width of less than or equal to 3 µm it is considered fine [53]. Percentage of scratches that were ‘parallel’ or ‘crisscross’ (*sensu* [53]) were also identified. For detailed information about classification and appropriate R methodology, see Strani *et al*. [53].

## 4. Systematic palaeontology

Dinosauria [54]

Sauropoda [55]

Titanosauriformes [56]

Somphospondyli [57]

Titanosauria [58]

Diamantinasauria [19]

### 4.1. *Diamantinasaurus matildae* [12]

**Figure 2.**
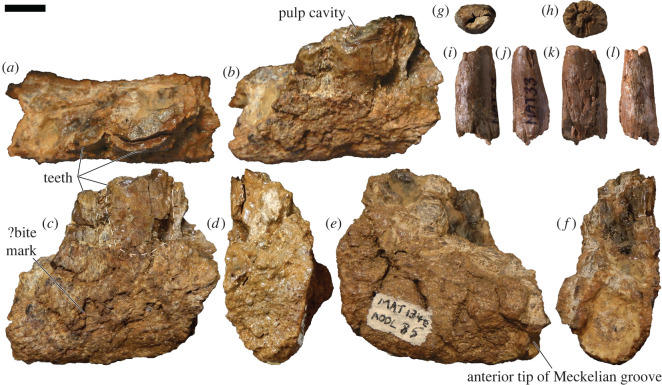
*Diamantinasaurus matildae* AODF 603 (AODL 85) dentary fragment and tooth. (*a–f*) Right dentary fragment (AODF 603) in occlusal (*a*), occluso–lingual (*b*), labial (*c*), mesial (*d*), lingual (*e*) and distal (*f*) views. (*g–l*) Tooth (AODF 603) in apical (*g*), basal (*h*), labial (*i*), mesial or distal (*j*), lingual (*k*) and mesial or distal (*l*) views. Scale bar = 10 mm.

*New paratype specimens:* AODF 603—right dentary fragment with teeth (figure 2*a–f*); isolated tooth crown (figure 2*g–l*).

*Locality:* AODL 85 (the ‘Matilda’ site), Elderslie Sheep Station, approximately 60 km west-northwest of Winton, west-central Queensland, Australia (figure 1).

*Horizon and age:* Winton Formation (Rolling Downs Group, Eromanga Basin; Cenomanian–lowermost Turonian [41,44].

*Description:* The preserved fragment of the right dentary (figure 2*a–f*; table 1) derives from near the anterior (mesial) end of the element. Both the presence of the anterior tip of the Meckelian groove (figure 2*e*), and the curvature of the fragment in occlusal view (figure 2*a*), support this interpretation. The ventral and lateral (=labial; figure 2*c*) surfaces of the dentary are both smoothly convex, whereas the less well-preserved medial (=lingual; figure 2*e*) surface is shallowly concave. Two broken teeth are preserved within the dentary (table 2). The more mesial tooth is more complete, despite being broken at approximately mid-crown height, and its labial surface is much more complete than the lingual one. The tooth appears to have been subjected to labiolingual compression, which has caused the enamel on the lingual surface to be displaced towards the labial surface. Its labial surface also shows compression in the form of a presumed bite mark. On the labial surface, the enamel and dentine combined are 2 mm thick in cross-section. The labial surface is flat to slightly convex. Dental carinae, if present, are not preserved. The enamel appears smooth throughout.

**Table 1.** Measurements (in millimetres) of the right dentary fragment of *Diamantinasaurus matildae*.

specimen no.	site	anteroposterior length	dorsoventral height	mediolateral breadth
AODF 603	AODL 85	49*	43.5*	22*

**Table 2.** Measurements (in millimetres) of sauropod tooth crowns from the Winton Formation. SI, slenderness index (apicobasal length/mesiodistal width of crown); CI, compression index (labiolingual breadth/mesiodistal width of crown). An asterisk (*) indicates an approximate measurement; a dagger (†) indicates a measurement taken on an incompletely preserved specimen; a double dagger (‡) indicates a tooth preserved within a dentulous element. Tooth roots were not included in any of the measurements presented here.

specimen no.	site	apicobasal height	mesiodistal length	labiolingual breadth	SI	CI
AODF 603^‡^	AODL 85	17.5*	16.2*	8.4*	—	—
AODF 603	AODL 85	23.5*	10.4*	8.5*	—	—
AODF 2298	AODL 127	27.0*	11.4*	10.5*	—	—
AODF 963	AODL 270	31.66	15.62	11.43	2.03	0.73
AODF 984	AODL 270	40.55	15.70	12.10	2.58	0.77
AODF 985	AODL 270	41.94	16.74	12.26	2.51	0.73
AODF 1285	AODL 270	23.03^†^	12.34^†^	10.62^†^	—	—
AODF 1286	AODL 270	34.68	13.25	14.46	2.62	1.09
AODF 1288	AODL 270	19.70^†^	12.15^†^	8.89^†^	—	—
AODF 1290	AODL 270	7.89^†^	—	—	—	—
AODF 1389	AODL 270	root only				
AODF 1531	AODL 270	39.10	13.60	9.60	2.88	0.71
AODF 1668	AODL 270	root only				
AODF 1669	AODL 270	15.72^†^	9.68^†^	—	—	—
AODF 1670	AODL 270	root only				
AODF 2291	AODL 270	24.29	12.06	10.49	2.01	0.87
AODF 2292	AODL 270	34.71	17.29	13.08	2.01	0.76
AODF 2293	AODL 270	18.63	9.12	8.83	2.04	0.97
AODF 2294	AODL 270	31.51	15.73	12.72	2.00	0.81
AODF 2295	AODL 270	28.01	13.45	12.38	2.08	0.92

The isolated, incomplete tooth crown (figure 2*g–l*) is missing its tip and root. Overall, the crown appears to show a low degree of lingual curvature (figure 2*j,l*). The labial, mesial and distal surfaces are smoothly convex along their lengths (figure 2*i,j*,*l*); by contrast, the lingual surface is mesiodistally convex at the base, but flat to shallowly concave near the apex (figure 2*k*). Consequently, the basal cross-section of the tooth is essentially circular (figure 2*h*), whereas the apical one is D-shaped (figure 2*g*). No enamel wrinkling, dental carinae or denticles can be observed, although in the case of the carinae this might be a consequence of non-preservation, rather than genuine absence. In apical view, the pulp cavity is minuscule; by contrast, the pulp cavity is prominent in basal view (7 mm mesiodistally × 4 mm labiolingually). The thinner enamel and thicker dentine layers together are 2 mm thick. Radial striations that extend between both layers are visible in apical and basal cross-sections (figure 2*g*,*h*).

### 4.2. *Diamantinasaurus matildae* [12]

**Figure 3.**
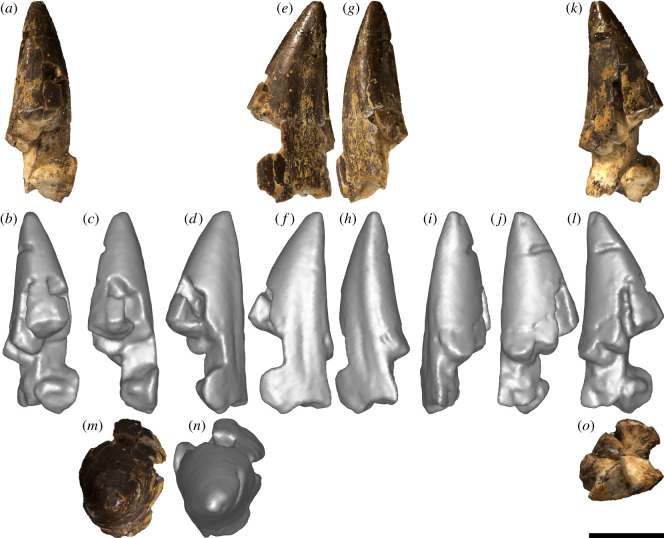
*Diamantinasaurus matildae* AODF 2298 (AODL 127) tooth (possibly from the same individual as AODF 836). (*a–o*) Right upper tooth in mesial (*a*,*b*), mesiolabial (*c*), labial (*d*), distolabial (*e*,*f*), distal (*g*,*h*), distolingual (*i*), lingual (*j*), mesiolingual (*k*,*l*), apical (*m*,*n*) and basal (*o*) views. (*a*), (*e*), (*g*), (*k*), (*m*) and (*o*) are photographs; all other images are screenshots of digital models. Scale bar = 10 mm.

*Tentatively referred specimen:* AODF 2298—partial tooth crown (figure 3*a–o*) from quadrant I9 of the ‘Alex’ Site, possibly from the same individual as AODF 836.

*Locality:* AODL 127 (the ‘Alex’ site), Belmont Station, approximately 60 km northeast of Winton, Central West Queensland, Australia (figure 1).

*Horizon and age:* Winton Formation (Rolling Downs Group, Eromanga Basin; Cenomanian–lowermost Turonian [41,44].

*Description:* A single partial tooth (figure 3*a–o*; table 2) was discovered in the quadrant (I9) immediately east of that which produced the atlas (I8) of AODF 836, a referred specimen of *D. matildae* [16,19]. This tooth is tentatively considered to be part of the same *Diamantinasaurus* individual. The asymmetry and subtle ‘twist’ of the tooth suggests that it was situated near the symphyseal margin [59]; thus, it is either a premaxillary tooth or a mesially situated dentary tooth. The surface of the tooth that preserves a shallow groove (overlap facet *sensu* Wilson & Sereno [57]) is interpreted herein as the distal one (figure 3*f*), meaning that this tooth is either from the right premaxilla or the left dentary.

The crown is incomplete basally but almost complete apically; only the absolute tip of the tooth has been lost, presumably broken during collection. Broadly speaking, the tooth is conical. The labial surface (figure 3*c*) is convex apicobasally, whereas the lingual surface (figure 3*i*) is shallowly concave apicobasally; consequently, the degree of lingual curvature is slight. The extent of the labial and lingual surfaces is uneven, the former being greater than the latter. In part, this is because the labial surface is strongly convex mesiodistally, whereas the lingual surface is only weakly so. However, this discrepancy is enhanced by the slight offset between the two surfaces, which is probably a reflection of the position of the tooth in the jaw.

In cross-section (figure 3*o*), the tooth is broadly D-shaped, albeit with the straight side of the ‘D’ somewhat convex. Although no mesial carina is present, the mesial junction between the labial and lingual surfaces is somewhat pronounced (figure 3*k*,*l*). On the distal margin, the labial and lingual surfaces merge more smoothly, such that there is no distal carina; however, the presence of a groove immediately labial to this junction gives the impression of a broad ridge (figure 3*d–h*). This groove appears to have been caused by pressure from an adjacent tooth, an interpretation supported by the texture of the enamel: within and around the groove, the enamel is roughened, whereas across the rest of the tooth it is smooth. Thus, this structure probably corresponds to an overlap facet. Similar grooves were observed on the carinae of sauropod tooth crowns from Lightning Ridge (AM F6670, AM F126713, LRF 1702 and LRF1519; [22]). The enamel is approximately 300 µm thick near the mesial margin (as measured along a broken surface with digital callipers).

### 4.3. ?Diamantinasauria indet

**Figure 4.**
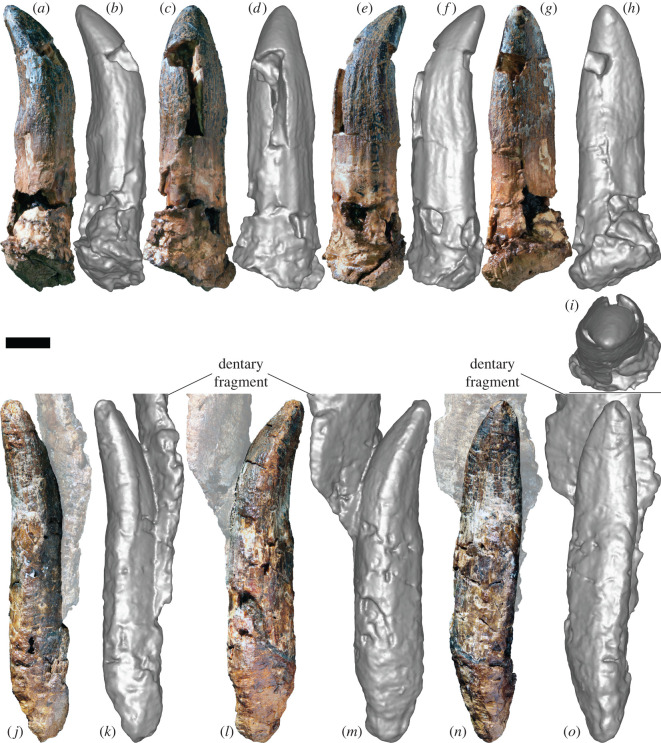
Titanosauria (?Diamantinasauria) indet. teeth AODF 963 and 1531 (AODL 270). (*a–i*) Left dentary tooth (AODF 963) in mesial (*a*,*b*), labial (*c*,*d*), distal (*e*,*f*), lingual (*g*,*h*) and apical (*i*) views. (*j–o*) Right dentary tooth attached to possible dentary fragment (AODF 1531) in distal (*j*,*k*), mesial (*l*,*m*) and lingual (*n*,*o*) views. (*a*), (*c*), (*e*), (*g*), (*j*), (*l*) and (*n*) are photographs; all other images are screenshots of digital models. Scale bar = 10 mm.

**Figure 5.**
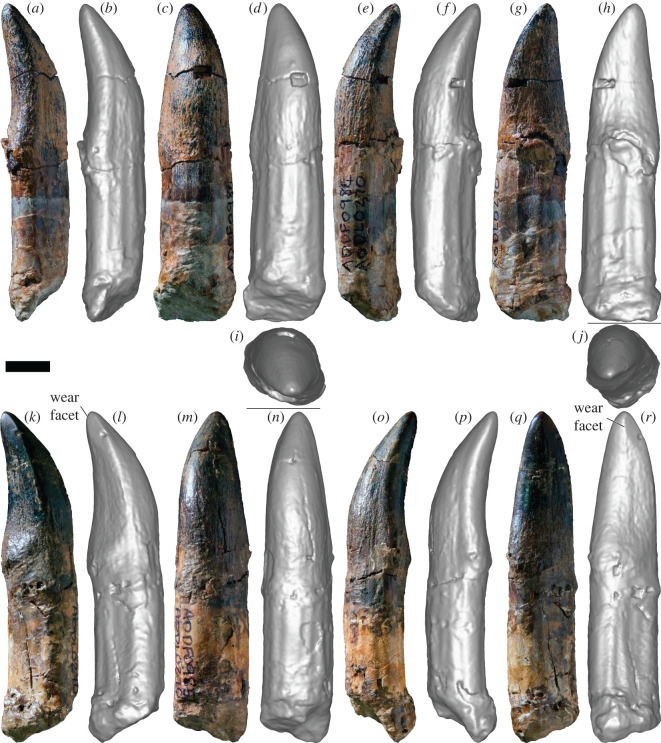
Titanosauria (?Diamantinasauria) indet. teeth AODF 984 and 985 (AODL 270). (*a–o*) Right dentary tooth (AODF 984) in distal (*a*,*b*), labial (*c*,*d*), mesial (*e*,*f*), lingual (*g*,*h*) and apical (*i*) views. (*j–r*) Left dentary tooth (AODF 985) in apical (*j*), mesial (*k*,*l*), labial (*m*,*n*), distal (*o*,*p*) and lingual (*q*,*r*) views. (*a*), (*c*), (*e*), (*g*), (*k*), (*m*), (*o*) and (*q*) are photographs; all other images are screenshots of digital models. Scale bar = 10 mm.

**Figure 6.**
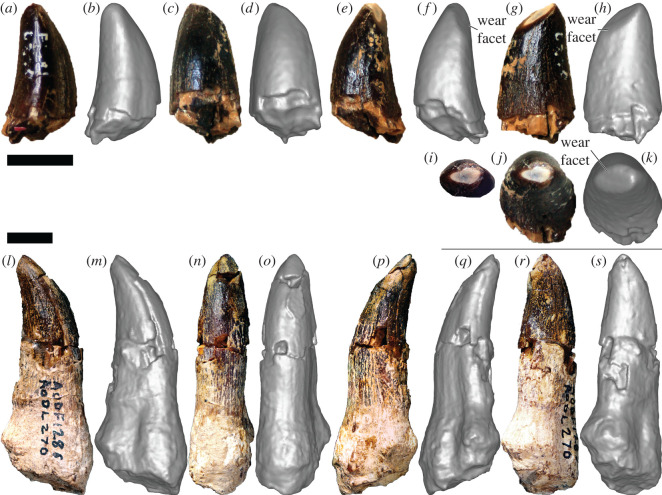
Titanosauria (?Diamantinasauria) indet. teeth AODF 1285 and 1286 (AODL 270). (*a–k*) Right dentary tooth (AODF 1285) in distal (*a*,*b*), labial (*c*,*d*), mesial (*e*,*f*), lingual (*g*,*h*) and apical (*i*–*k*) views. (*l–s*) Left dentary tooth (AODF 1286) in mesial (*l*,*m*), labial (*n*,*o*), distal (*p*,*q*) and lingual (*r*,*s*) views. (*a*), (*c*), (*e*), (*g*), (*i*), (*j*), (*l*), (*n*), (*p*) and (*r*) are photographs; all other images are screenshots of digital models. Scale bars = 10 mm.

**Figure 7.**
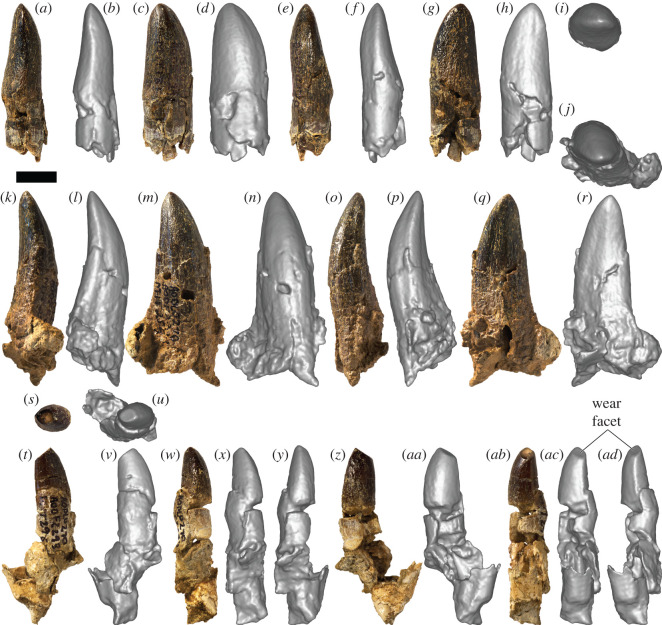
Titanosauria (?Diamantinasauria) indet. teeth AODF 2291, 2292 and 2293 (AODL 270). (*a–i*) Right dentary tooth (AODF 2291) in distal (*a*,*b*), labial (*c*,*d*), mesial (*e*,*f*), lingual (*g*,*h*) and apical (*i*) views. (*j–r*) Right dentary tooth (AODF 2292) in apical (*j*), distal (*k*,*l*), labial (*m*,*n*), mesial (*o*,*p*) and lingual (*q*,*r*) views. (*s*–*ad*) Left dentary tooth (AODF 2293) in apical (*s*,*u*), lingual (*t*,*v*), mesiolingual (*w*,*x*), mesial (*y*), labial (*z,aa*), distolabial (*ab*,*ac*) and distal (*ad*) views. (*a*), (*c*), (*e*), (*g*), (*k*), (*m*), (*o*), (*q*), (*s*), (*t*), (*w*), (*z*) and (*ab*) are photographs; all other images are screenshots of digital models. Scale bar = 10 mm.

**Figure 8.**
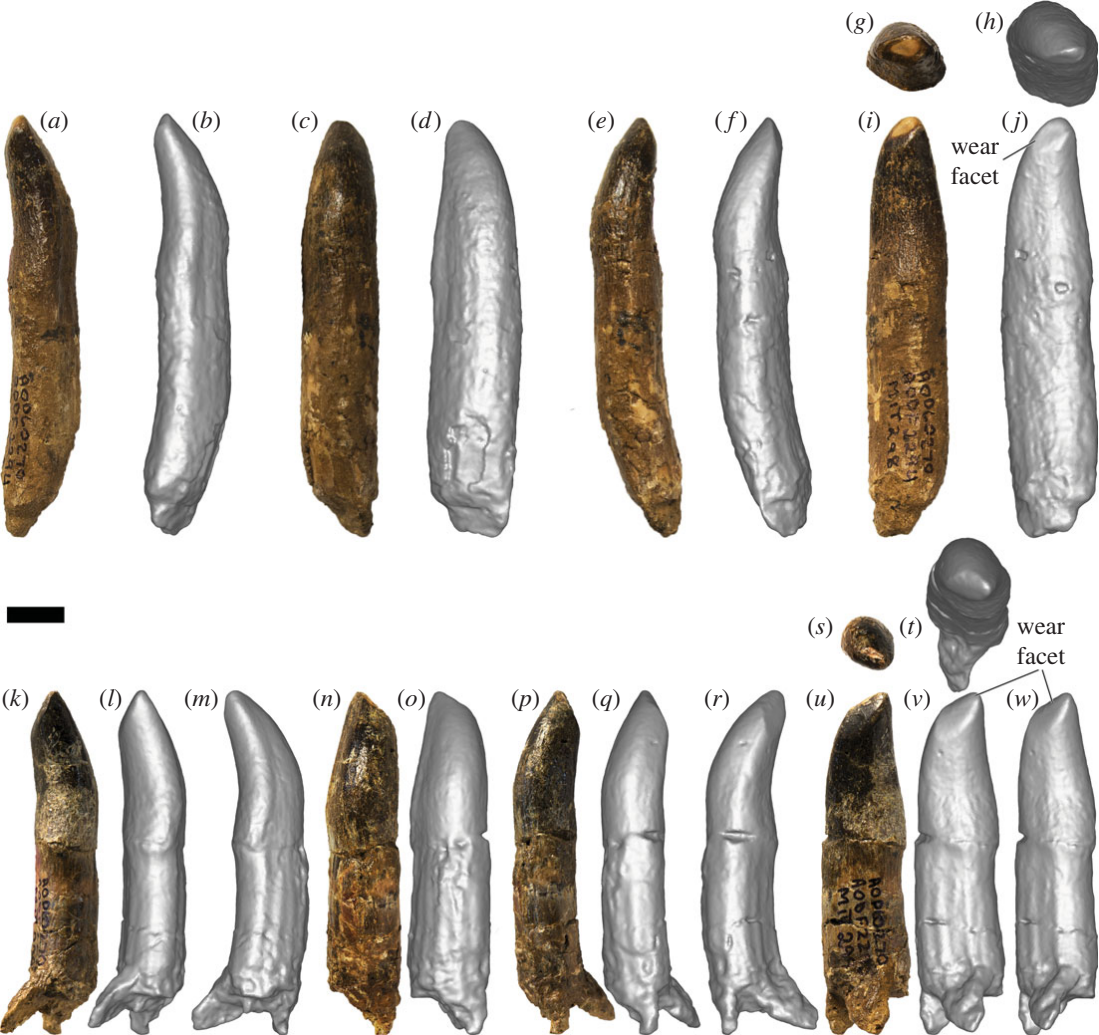
Titanosauria (?Diamantinasauria) indet. teeth AODF 2294 and 2295 (AODL 270). (*a–j*) Right dentary tooth (AODF 2294) in distal (*a*,*b*), labial (*c*,*d*), mesial (*e*,*f*), apical (*g*,*h*) and lingual (*i*,*j*) views. (*k–w*) Right dentary tooth (AODF 2295) in distolingual (*k*,*l*), distal (*m*), labial (*n*,*o*), mesiolabial (*p*,*q*), mesial (*r*), apical (*s*,*t*), mesiolingual (*u*,*v*) and lingual (*w*) views. (*a*), (*c*), (*e*), (*g*), (*i*), (*k*), (*n*), (*p*), (*s*) and (*u*) are photographs; all other images are screenshots of digital models. Scale bar = 10 mm.

*Specimens:* AODF 963—slightly worn tooth crown with partial root (figure 4*a–i*); AODF 984—slightly worn tooth crown with root (figure 5*a–i*); AODF 985—slightly worn tooth crown with root (figure 5*j–r*); AODF 1285—tooth crown with prominent wear facet (figure 6*a–k*); AODF 1286—incomplete tooth crown (figure 6*l–s*); AODF 1288—fragmentary tooth crown; AODF 1290—fragmentary tooth crown; AODF 1389—fragmentary tooth root; AODF 1531—slightly damaged tooth crown and root with associated dentulous element fragment (figure 4*j–o*); AODF 1668—fragmentary tooth crown; AODF 1669—fragmentary tooth with associated dentulous element fragment; AODF 1670—fragmentary tooth crown; AODF 2291—tooth crown with partial root (figure 7*a–i*); AODF 2292—tooth crown with partial root (figure 7*j–r*); AODF 2293—partial tooth crown with prominent wear facet and partial root (figure 7*s*–*ad*); AODF 2294—complete tooth with prominent wear facet (figure 8*a–j*); AODF 2295—almost complete tooth with prominent wear facet (figure 8*k–w*).

*Locality:* AODL 270 (the ‘Mitchell’ site), Elderslie Sheep Station, approximately 60 km west-northwest of Winton, west-central Queensland, Australia (figure 1).

*Horizon and age:* Winton Formation (Rolling Downs Group, Eromanga Basin; Cenomanian–lowermost Turonian [41,44].

*Description and comparisons:* The morphology of the sauropod teeth from the ‘Mitchell’ site is essentially uniform (figures 4–8; table 2). The most complete exemplars (AODF 963, AODF 984, AODF 985, AODF 1531 and AODF 2294) form the basis for most of the description herein, although less complete specimens (AODF 1285, AODF 2293 and AODF 2295) are discussed in the section on tooth wear. Given that they closely resemble the teeth associated with the two skeletons of *Diamantinasaurus*, but are better preserved, we also base our comparisons with other sauropods on the ‘Mitchell’ site teeth.

AODF 1531 is preserved in connection with a fragment of bone that appears to be the lateral wall of the right dentary or left premaxilla/maxilla (figure 4*i–o*). If this interpretation is correct, then the placement of the remaining teeth can be inferred. AODF 984, AODF 1285, AODF 1288, AODF 2291, AODF 2292, AODF 2294 and AODF 2295 are essentially identical to AODF 1531, implying that they are also from the right dentary or left premaxilla/maxilla. AODF 985 and AODF 2293 are each effectively mirror images of these teeth (with AODF 2293 substantially smaller), so they presumably derive from the left dentary or right premaxilla/maxilla (with AODF 2293 probably being quite distal). AODF 963 and AODF 1286 are similar to AODF 985, albeit with slightly more pronounced lingual curvature, and are herein interpreted as being from the left dentary/right premaxilla/maxilla as well. For convenience and brevity, we will assume from this point on that the teeth all derive from dentaries (an interpretation supported by the position of the wear facets in several of the teeth that preserve them), although we note that this might be shown to be incorrect in future.

The only complete tooth roots, those of AODF 1531 and AODF 2294, are slightly longer than the crowns. The other teeth with nearly complete crowns have roots approximately equal in length. In cross-section, the root of each tooth is elliptical, being slightly longer mesiodistally than labiolingually. Along the length of the root, pronounced apicobasal ridges are present. All are fairly well-defined, but the most prominent on several teeth are those on the lingual surface (e.g. AODF 984 (figure 5*h*), AODF 985 (figure 5*r*), AODF 2295 (figure 8*v–w*)). The presence of weakly developed apicobasal ridges on the root was recently proposed as a synapomorphy of Turiasauria [60]. Given the clear differences between the AODL 270 teeth and the broad, heart-shaped teeth of turiasaurians [61,62], we regard the presence of well-developed apicobasal ridges on the root as a possible diagnostic feature of the AODL 270 teeth and thus, tentatively, a local synapomorphy of Diamantinasauria.

The root–crown boundary of the teeth shows essentially no constriction, as in neosauropods generally [63,64]. The crown bulges slightly in its basal third, before tapering towards the apex, albeit asymmetrically: viewed lingually or labially, the distal margin of the tooth is straighter than the mesial one. In both labial and lingual views, the AODL 270 tooth crowns are compressed-cone-chisel-shaped *sensu* Calvo [65]. In cross-section, the base of each crown is roughly D-shaped, with the straighter side of the ‘D’ being the lingual surface. The labial surface is smoothly convex both apicobasally and mesiodistally. By contrast, the lingual surface is shallowly concave apicobasally and gently convex mesiodistally, with the apex of this convexity closer to the distal margin than the mesial one. There is no midline convexity on the lingual surface, thereby setting the AODL 270 teeth apart from most eusauropods outside of the diplodocoid and somphospondylan radiations [1,64]. The teeth from AODL 270 appear to be slightly ‘twisted’, albeit less so than is typical of brachiosaurid maxillary teeth [2,66]. In large part, the apparent ‘twist’ of the teeth stems from the fact that the distal region of the base of the lingual surface is more expanded than the mesial one. The teeth of *Euhelopus* show similar asymmetry: the basally positioned distal lingual buttress is always more strongly developed than the mesial one [67,68]. The intersection between the labial and lingual surfaces is marked on each side by a very weakly defined carina, as is common in somphospondylans [64,69], with the mesial carina slightly more pronounced than the distal one, and the latter characterized by an overlap facet. Both the mesial and distal margins also lack denticles, distinguishing the AODL 270 teeth from those of many non-titanosaurian macronarians, including *Europasaurus* [70], *Giraffatitan* [26], *Vouivria* [71], *Mongolosaurus* [69,72] and *Phuwiangosaurus* [73], as well as some titanosaurs, including the lognkosaurian *Quetecsaurus* [74]. Although the teeth of the early branching somphospondylan *Ligabuesaurus* were originally described as possessing denticles [75], more recent appraisal has suggested that this is incorrect [76,77]. The carinae of the teeth of the lithostrotian titanosaur *Tapuiasaurus* are interrupted by relatively regularly spaced notches (‘grooves’ *sensu* Zaher *et al*. [78]); similar structures are not evident on the teeth from AODL 270.

The slenderness index (SI: apicobasal length of crown divided by maximum mesiodistal width [63]) for all complete tooth crowns falls between 2.00 and 2.88 (table 2). In this regard, these teeth are intermediate between the ‘spatulate’ teeth of taxa such as *Camarasaurus* [79–81] and *Euhelopus* [67,82] and the ‘chisel-like’ teeth of many titanosaurs [65] (see also Barrett *et al*. [1], Chure *et al*. [2] and Mocho *et al*. [6]). Similar SI values have been obtained for the teeth of *Europasaurus* [64], Brachiosauridae [2], early branching somphospondylans [83], the early diverging titanosaurs *Choconsaurus* [84] and *Sarmientosaurus* [85], and sauropod tooth morphotypes ‘B’, ‘C’ and ‘D’ from the Griman Creek Formation [22].

The tooth enamel can be observed in cross-section in AODF 984 and is 1 mm thick around the entire circumference of the crown. Although the external surface of the enamel of the AODL 270 teeth is smooth, this appears to be in part caused by a very fine ironstone patina that has infilled multiple shallow, predominantly longitudinal, enamel wrinkles. Thus, as in eusauropods generally [4,57], the teeth from AODL 270 are characterized by wrinkled enamel. On the labial surface, near the distal carina, several parallel, longitudinal grooves are present. Similar variation in enamel texturing was reported in *Nemegtosaurus*, wherein the tooth crowns are finely wrinkled for the most part, with longitudinal ridges near the crown base [86]. In other somphospondylan taxa in which these have been documented (e.g. *Huabeisaurus*), these grooves tend to be present along the full length of the crown, on both the labial and lingual surfaces [83].

Prominent wear facets are present on at least five teeth in the sample (AODF 985, AODF 1285, AODF 2293, AODF 2294 and AODF 2295), with at least three others having weakly defined wear facets (AODF 963, AODF 984 and AODF 1531). The wear facet on AODF 985 is situated mesiolingually and does not impact the apex, whereas those on AODF 1285, AODF 2294 and AODF 2295 are also situated mesiolingually, but are sufficiently extensive that they have affected the apex. The wear facet on AODF 985 is small and does not extend below the enamel. It is quite high-angled, less than 10° relative to the apicobasal long axis. By contrast, the wear facets on AODF 1285, AODF 2294 and AODF 2295 are large and have extended sufficiently basally that the dentine beneath the enamel is visible in each tooth (less clearly in AODF 2295). Moreover, these wear facets are lower-angled (approx. 45° relative to the apicobasal long axis) than that in AODF 985. Mesiolingual wear facets are not uncommon in titanosauriform sauropod teeth, even without an accompanying distal or distolingual wear facet. Examples of titanosauriform teeth that solely preserve a mesiolingual facet include: at least one specimen (MB.R.2181.23.9) of *G. brancai* [4,26]; *Abydosaurus mcintoshi* [2]; one of the possible brachiosaurid teeth (JAzar 1) from the Early Cretaceous of Lebanon [87]; an isolated tooth (MCF-PVPH-744) of *Ligabuesaurus leanzai* [75,88]; some of the teeth of *Choconsaurus baileywillisi* [84]; and several titanosaurian teeth from the latest Cretaceous Anacleto and Allen formations of Argentina [89]. Thus, such wear facets have been reported in both non-titanosaurian and titanosaurian titanosauriforms, albeit much more rarely in the latter. These wear facets observed in the AODL 270 teeth match those of indeterminate titanosauriform dentary teeth designated as ‘Type 3’ by Saegusa & Tomida [90].

The wear facet on AODF 2293 is different from the other ‘Mitchell’ site teeth in that it is situated distolabially and affects the apex. A similar wear facet was observed in the sole preserved tooth of the brachiosaurid *Soriatitan golmayensis* [91]. Based on the position of the wear facet in AODF 2293, it is likely that it is from the opposite jaw to the other teeth with wear facets from AODL 270 (i.e. if they are from the lower jaw as we interpret herein, AODF 2293 is from the upper jaw), as the distolabial position of the wear facet in AODF 2293 is opposed to the mesiolingual position of the wear facets in AODF 985, AODF 1285, AODF 2294 and AODF 2295.

### 4.4. SEM microwear analysis of teeth from AODL 270

**Figure 9.**
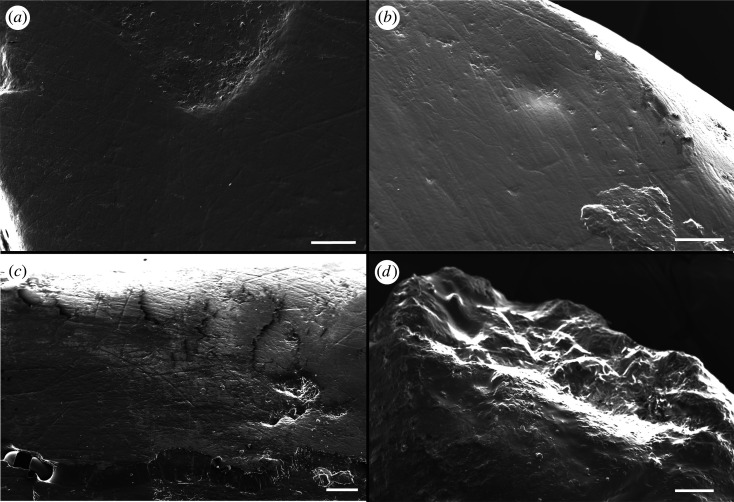
Scanning electron micrographs of microwear features preserved on the Titanosauria (?Diamantinasauria) indet. teeth from the Winton Formation. (*a*) AODF 1285 displaying the roughened dentine within the enamel wear facet. (*b*) AODF 963 featuring the large ‘gouge’ like pits. (*c*) AODF 985 showing mostly parallel scratches with some cross-scratching. (*d*) AODF 1531 displaying roughened apical texture. Scale bar = 200 µm in (*a*–*c*) = 500 µm in (*d*).

Three out of the five sauropod teeth examined under the SEM preserve microwear features (AODF 963, AODF 985 and AODF 1285) (table 3). The dentine is exposed in AODF 1285 (figure 9*a*); however, this section was not subjected to microwear analysis, as the dentine wears faster than enamel and can skew the analysis [92]. All three teeth show similar scratch patterns but differ in pit frequencies. Both fine (width less than or equal to 3 µm) and coarse scratches are present, with the latter being the most frequent. These scratches are mostly parallel across the sample, with limited cross-scratching; AODF 985 shows the most cross-scratching of the teeth sampled (figure 9*c*). Both AODF 963 and AODF 985 have a higher frequency of large pits (width greater than or equal to 8 µm), whereas AODF 1285 preserves a higher frequency of small pits. The large pits preserved on AODF 963 are often sub-circular in shape and have an irregular margin (figure 9*b*).

**Table 3.** ‘MicroWeaR’ results of the microwear analysis applied to the Winton sauropod teeth. Abbreviations: #Pits, number of pits; #Sp, number of small pits; #Lp, number of large pits; %P, percentage of pits; P, ratio of pits mm^−^^2^; #Scratches, number of scratches; #Fs, number of fine scratches; #Cs, number of coarse scratches; S, ratio of scratches mm^−^^2^; #Ps, number of paired parallel scratches; #Xs, number of cross-scratches. All measurements are in micrometres.

AODF 963	#Pits	#Sp	#Lp	%P	P	#Scratches	#Fs	#Cs	S	#Ps	#Xs
count	49	—	49	44	4	63	2	61	5.8	41	18
mean length	25.9	—	25.9	—	—	243.3	274.73	243	—	—	—
mean width	12	—	12	—	—	9.9	1.5	10.3	—	—	—
AODF 985	#Pits	#Sp	#Lp	%P	P	#Scratches	#Fs	#Cs	S	#Ps	#Xs
count	100	37	63	24	2.6	313	38	275	7.1	500	139
mean length	12.2	7	12.8	—	—	264.2	143.1	292.9	—	—	—
mean width	6.5	4.3	7.2	—	—	6	1.7	6.8	—	—	—
AODF 1285	#Pits	#Sp	#Lp	%P	P	#Scratches	#Fs	#Cs	S	#Ps	#Xs
count	94	82	12	47	5.875	106	38	68	6.6	107	30
mean length	7.3	6.1	15	—	—	161.6	111.9	188.6	—	—	—
mean width	2.3	1.7	3.5	—	—	3.8	1.7	4.9	—	—	—

There is a possibility that the microwear features observed in the SEM analysis were caused by non-dietary factors such as post-mortem abrasion or transport. Both AODF 984 and AODF 1531 show signs of taphonomic damage, as the apical surface is roughened, which is potentially why no microwear features are preserved (figure 9*d*). By contrast, given the relative microwear consistency and a lack of roughened sections in the other three teeth (AODF 963, AODF 985 and AODF 1285), these features are much more likely to be dietary in nature and not formed by other environmental means.

Given the similarity between all three teeth in overall morphology and SI values, the differences in microwear are potentially related to the position in the jaw: AODF 1285 pertains to the right dentary, and AODF 963 and AODF 985 pertain to the left dentary. The wear facet in AODF 1285 is also angled lower (approx. 45°) than that in the other two teeth (less than 10°), which further supports this differentiation in tooth position [93]. Although differences in microwear features have been noted between left and right premaxillary teeth from a single *Diplodocus* specimen [94], these differences were not held up as representing a left/right trend.

## 5. Discussion

### 5.1. Phylogenetic placement of the Winton Formation sauropod teeth

The sauropod teeth described herein are referable to Eusauropoda as they are spatulate, have wrinkled enamel texture, and show evidence of crown-to-crown occlusion [57,63]. Referral to Turiasauria is precluded because the teeth are not heart-shaped, nor do they possess an apicobasal bulge/midline ridge within the lingual concavity [6,60–62,95,96]. The fact that the root–crown boundary shows essentially no constriction—in contrast to the teeth of early branching eusauropods like *Bagualia* [97], *Bellusaurus* [98,99], *Chebsaurus* [100,101], *Mamenchisaurus* [102,103], *Omeisaurus maoianus* [104], cf. *Patagosaurus* [4] and *Shunosaurus* [105]—aligns the Winton Formation teeth with Neosauropoda [63,64]. Within Neosauropoda, the teeth can be excluded from Diplodocoidea as they are neither narrow-crowned nor peg-like [57,94,106]. They are also easily differentiable from the strongly spatulate teeth of the earliest branching macronarians [63,65], such as *Camarasaurus* [80,107] and *Europasaurus* [70]. Thus, the available evidence indicates that the Winton Formation sauropod teeth described herein are referable to Titanosauriformes.

Within Titanosauriformes, the teeth show some similarities with the teeth of brachiosaurids; however, they can be readily differentiated as they do not show the distinct ‘twist’ seen in *Abydosaurus*, *Giraffatitan* or *Vouivria* [2,66,71]. Similarly, they do not conform with the narrow-crowned tooth morphology displayed by nearly all post-Turonian titanosaurs (see below). Instead, the Winton Formation sauropod teeth most closely resemble those of the non-titanosaurian somphospondylan *Ligabuesaurus leanzai* [75,77], as well as the early branching titanosaurs *Choconsaurus baileywillisi* [84] and *Sarmientosaurus musacchioi* [85]. This implies that they pertain to either a non-titanosaurian somphospondylan, or to an early branching titanosaurian.

The association of two of these teeth with the type and referred specimens of *Diamantinasaurus matildae*, respectively, and their morphological similarities with the AODL 270 ‘Mitchell’ site teeth, implies that the Winton Formation teeth all pertain to diamantinasaurians, and therefore to early branching titanosaurs [19]. However, given that the early branching somphospondylan *Wintonotitan wattsi* is also known from the Winton Formation [12,14], the referral of the AODL 270 teeth to Diamantinasauria cannot be viewed as unequivocal—unless the recent recovery of *Wintonotitan wattsi* as a member of Diamantinasauria in some of the analyses conducted by Hocknull *et al*. [13] proves correct. If *Wintontotitan* is a diamantinasaurian, then all diagnostic sauropod material presently known from the Winton Formation is referable to Diamantinasauria. However, future research might demonstrate that a greater diversity of sauropods (at a higher phylogenetic level) was present in the Winton Formation than the evidence presently suggests, hence our cautionary referral of the AODL 270 teeth to that clade.

### 5.2. Wear facets

The lack of wear facets on most of the AODL 270 sauropod teeth suggests that they were replacement teeth detached from a jaw, rather than active teeth that were dislodged or shed. However, virtually all of the teeth preserve a distal overlap facet, indicating pressure from the crown of their mesial neighbour. Initially, we interpreted this as an indication that the teeth were erupted or nearly so. However, in a specimen of *Camarasaurus*, replacement teeth in adjacent tooth positions overlap one another mesiodistally, with no hard tissue defining individual alveoli deeper than a few centimetres (JA Whitlock 2022, personal communication). Thus, we suggest that the teeth of this diamantinasaurian sauropod overlapped within the crypt during development, at least in the latter stages. Only five of the teeth preserve wear facets: one preserves a small, high-angle plane, whereas the other four each host a larger, low angle plane that cross-cuts the enamel and dentine layers. These teeth at least appear to have been in use perimortem, and the nature of the wear facet implies either that the teeth of its owner occluded precisely (rather than interlocking and creating high-angle wear facets mesially and distally), and/or that the teeth were employed to acquire particularly tough foodstuffs.

The SEM analysis of the Winton Formation sauropod teeth reveals a higher proportion of scratches than pits on the wear facets. This implies that the sauropod to which these teeth belonged fed at mid-height (1–10 m): the relative abundance of pits suggests against a high browse height (greater than 10 m; [94,108,109]). The higher frequency of coarse scratches on the teeth, coupled with the presence of some cross-scratching, suggests a diet of hard foodstuffs [94,110], similar to that predicted for *Sarmientosaurus* [85]. However, it is possible the higher frequency of coarse parallel scratches in the sample is a consequence of crown–crown contact rather than dietary preference, as in *Camarasaurus* and *Giraffatitan* [94].

The only other Australian sauropod teeth for which microwear features have been examined are those from the Griman Creek Formation at Lightning Ridge [22]. Two tooth morphotypes were shown to have varying proportions of features preserved: morphotype A has large pits with fine scratches that show no signs of cross-scratching, whereas morphotype D shows several gouges (larger than pits) alongside small pits with coarse scratches that frequently cross-scratched. These were tentatively interpreted as indicating differentiation of diets between two potential species, with morphotype A feeding at mid-height (1–10 m) on softer foodstuffs than morphotype D, a ground level feeder (0–1 m; [22]). The inferred diet of the Winton Formation sauropod differs from each of the Lightning Ridge sauropod tooth morphotypes, since it is interpreted as a mid-height feeder feeding on hard foodstuffs. Although this might imply a palaeoecological difference between the two geographical regions (the formations are, at least in part, roughly coeval), the fact that the palaeoflora from Lightning Ridge remains undescribed precludes a comparison between potential food sources at this time [33]. However, the Winton Formation flora has been described, meaning that the potential diet of the Winton Formation sauropods can be constrained.

### 5.3. Dietary options for early Late Cretaceous sauropod dinosaurs of northeast Australia

The ‘upper’ Winton Formation preserves an impressive array of plant macrofossils and microfossils pertaining to a diversity of higher-level taxa. The flora has been reported to comprise more than 50 plant macrofossil taxa [111], although a subsequent publication stated that fewer than 30 species were present and consequently characterized the flora as being far less diverse than similarly aged floras from comparable palaeolatitudes [45]; however, if the former figure (50 species) is used, this discrepancy is eliminated for some coeval floras, and diminished for others.

In the Winton Formation flora, hepatophytes are represented by gemmae assigned to *Marchantites marguerita* [112], whereas horsetails are represented by *Equisetites* sp. macrofossils [111]. Ferns are quite diverse and abundant, with osmundaceans (*Phyllopteroides macclymontae* [111,113], *Cladophlebis* sp. [113]), gleicheniaceans (*Microphyllopteris* sp. cf. *M. gleichenoides* [113]) and tempskyaceans (*Tempskya judithae* [114]) all present, alongside species of uncertain phylogenetic position (e.g. *Sphenopteris* sp. cf. *S. warragulensis* [113]). Ginkgos are represented by *Ginkgo wintonensis* [113], cycads possibly by *Pterostoma hirsutus* [115] and bennettitaleans by *Otozamites* cf. *bengalensis* and *Ptilophyllum* sp. [111]. However, the Winton Formation fossil flora is co-dominated by coniferophytes and angiosperms [111,113]. Coniferophytes are common and diverse, with cupressaceans (*Austrosequoia wintonensis* [50]), araucariaceans (several *Araucaria* morphotypes [111,113] and *E. microcarpa* [51]) and podocarpaceans (*Protophyllocladoxylon owensii* [116]) all present. All of the angiosperms present are referable to Magnoliopsida, with nine morphotypes referable to Fagales [111,113], and one to Laurales (*Lovellea wintonensis* [117]).

The most detailed assessments of sauropod diet to date have focused on the Upper Jurassic Morrison Formation of North America, either through analysis of the palaeoflora [118,119] or of the sauropods represented in the fauna [120–123]. Other studies have correlated Early Jurassic palaeofloral changes in South America with changes in sauropod faunal composition [97]. Whereas the clearest difference between Jurassic palaeofloras and that of the Upper Cretaceous Winton Formation is that the former lack angiosperms [124], one commonality is that coniferophytes are diverse and abundant [125]. Whether or not sauropods fed on angiosperms is unclear, since there is currently no direct evidence for sauropod dietary preferences in the fossil record [126]. However, wide-ranging studies on sauropod diet have highlighted gingkoes, coniferophytes and horsetails as likely sauropod food sources based on multiple lines of evidence [126–128], with araucariaceans the most likely food source among coniferophytes [118].

Both coniferophytes and ginkgoes would probably have had foliage and/or fruiting bodies within the 1–10 m height range, hypothesized here as the diamantinasaurian feeding envelope. Horsetails today achieve heights up to 2 m, so they too might have been on the menu. The relatively robust teeth of diamantinasaurian sauropods would have enabled them to procure parts of plants that were relatively hardy or strongly attached. Moreover, the wear facets present on several of the recovered teeth might have occurred through procurement of tough foodstuffs with the active dentition.

### 5.4. The Berriasian–Turonian sauropod body fossil record

The Early–early Late Cretaceous (Berriasian–Turonian) was a time of sauropod change worldwide [129], bookended by the decline of flagellicaudatans in Laurasia [106] and the rise to dominance of titanosaurs worldwide [64,66,130,131]. The opening of the Atlantic Ocean [132,133], the closure of the Tethys Ocean and the concomitant opening of the ‘Apulian route’ [134], and episodic inundation of various continents by inland seaways during this interval means that the nature and timing of this faunal turnover varied on different continents [135–137]. Below, we review the sauropod fossil record on each palaeocontinental region during this interval, with a particular emphasis on teeth and titanosauriforms.

*Australasia and Zealandia.* The oldest Cretaceous sauropod body fossils from Australasia + Zealandia are titanosauriforms from the upper Albian Toolebuc Formation and Allaru Mudstone of Queensland, Australia [8,17,138]. The upper Albian–Cenomanian Griman Creek Formation has produced titanosauriform and early branching titanosaur teeth [11,22,33], whereas the Cenomanian Winton Formation has produced evidence of both non-titanosaurian somphospondylans [7,12,14] and early branching titanosaurs [12,13,15,16,18,19,21]. So far, no rebbachisaurids have been found in Australasia + Zealandia, and it has been hypothesized that they never made it to these continents [19]. No sauropod specimens are known from Zealandian deposits stratigraphically older than the Campanian [139,140].

*Antarctica, Madagascar and India.* No Early Cretaceous sauropod body fossils have been reported from Antarctica, Madagascar or India. Early Late Cretaceous records are limited to indeterminate sauropod remains from the Cenomanian of both Madagascar [141] and India [142]. The Antarctic sauropod record is limited to a titanosaurian partial vertebra from late Campanian deposits [143].

*South America.* Berriasian–Valanginian deposits in Argentina have produced remains of dicraeosaurid and diplodocid diplodocoids [144–149], all characterized by narrow-crowned teeth and all ‘holdovers’ from latest Jurassic faunas. However, contemporaneous deposits in this region also host the oldest known putative titanosaur, *Ninjatitan* [150]. Berriasian–Hauterivian deposits in Brazil have also yielded putative titanosaurs, including *Triunfosaurus* [151,152], although these have more recently been regarded as non-titanosaurian somphospondylans [17,131]. In Argentina, Hauterivian–Barremian deposits have produced remains of titanosauriforms [153,154], but dicraeosaurids dominate the Barremian deposits [155–161]. Terminal Barremian deposits in Colombia have produced the titanosauriform *Padillasaurus*, originally described as a brachiosaurid by Carballido *et al*. [162] but reinterpreted as a somphospondylan by Mannion *et al*. [71], whereas upper Barremian–lower Aptian deposits in Argentina host rebbachisaurids [163]. The lithostrotian titanosaur *Tapuiasaurus*, which derives from Aptian-aged rocks in Brazil [59,78], has narrow-crowned, chisel-like teeth that are typical of derived titanosaurs.

Transitional Aptian–Albian deposits in both Brazil [164] and Argentina [165–167] are dominated by rebbachisaurids, although fragmentary remains belonging to titanosauriforms are also present in the Brazilian deposits [168], and the non-titanosaurian somphospondylan *Chubutisaurus* is present in Argentina [169–171]. Albian-aged deposits in Argentina show some variation. Lower Albian deposits in Neuquén Province have yielded rebbachisaurids and non-titanosaurian somphospondylans including *Ligabuesaurus* [75,77,88,172–174], whereas upper Albian deposits in Chubut Province have produced the lognkosaurian titanosaur *Patagotitan* [175,176], but no rebbachisaurids. Rebbachisaurids are represented in the Albian–Cenomanian of Brazil [177], and dominate Cenomanian–Turonian sauropod faunas across Argentina [178–188]. The latter deposits also preserve a variety of titanosaurs, including early branching forms, such as *Andesaurus*, *Epachthosaurus* and *Sarmientosaurus*, as well as lognkosaurians including *Argentinosaurus* [58,74,84,85,189–198]. Post-Turonian Cretaceous strata in South America lack rebbachisaurids, with their sauropod faunas dominated by lithostrotian titanosaurs [199–205].

Aside from *Tapuiasaurus* in Aptian deposits in Brazil, no pre-Turonian titanosaurs with narrow-crowned teeth are known from South America. A closer affinity between the sauropod faunas of northeast South America and those of northwest Africa, rather than those of southern South America, has been demonstrated [206], so this north–south differentiation within South America (i.e. titanosaurs with narrow-crowned teeth in the northeast but not the south) is perhaps unsurprising. Nevertheless, the absence of titanosaurs with narrow-crowned teeth in Argentina prior to the Turonian is quite striking, and suggests that some sort of environmental or topographic barrier might have existed between southwestern and northeast South America, albeit one that rebbachisaurids were able to cross.

*Africa and the Middle East.* Berriasian–Valanginian deposits in southern Africa have yielded remains of dicraeosaurid and diplodocid diplodocoids, as well as brachiosaurids and sauropods of uncertain affinity [207–209]. Early Cretaceous ('Neocomian') strata in Lebanon have produced teeth that appear to be referrable to Brachiosauridae [64,87]. All of these records represent ‘holdovers’ from the Late Jurassic, based on comparisons with the sauropod fauna of the Tendaguru Formation of Tanzania [210,211]. Hauterivian–Barremian deposits in Libya have yielded a single sauropod tooth, preserving the base of the crown and root, which was originally attributed to a camarasaurid by Le Loeuff *et al*. [212]. Mocho *et al*. [6] and Royo-Torres *et al*. [60] referred this tooth to Turiasauria, although Mannion [96] questioned this referral, arguing that it can only be assigned to Eusauropoda, whereas Holwerda [213] suggested that it pertains to a titanosauriform. Upper Hauterivian–Lower Barremian deposits in Croatia, which was then part of the Afro-Arabian continent, demonstrate that rebbachisaurids coexisted with titanosauriforms (most likely early-branching somphospondylans) at this time [214–216]. In Malawi, lithostrotian titanosaurs were established as early as the Aptian [217–222], with evidence that *Karongasaurus*, which has narrow, chisel-like teeth, lived alongside *Malawisaurus*, which has broader, compressed-cone-chisel-like teeth [222]. Aptian–Albian deposits in Niger are dominated by rebbachisaurids [223–226], although titanosauriforms (probably somphospondylans) were also present [227]. Aptian–Albian deposits in Cameroon preserve narrow-crowned sauropod teeth [228], possibly attributable to Somphospondyli [227]. A mid-Cretaceous (Aptian–Cenomanian) site in Tanzania has produced sauropod teeth that correspond to three distinct morphotypes, all attributed to the titanosaur *Mnyamawamtuka* [229]. Whereas one of these morphotypes is extremely narrow-crowned with high-angle wear facets (and therefore reminiscent of the teeth of derived lithostrotians), another is more robust and has a D-shaped cross-section, implying (as do the postcranial remains) that *Mnyamawamtuka* is an early-branching titanosaur [229]. Rebbachisaurids and somphospondylans coexisted during the early Albian in Tunisia [230–232] and the late Albian–early Cenomanian in Morocco [5,213,227,233–237]. Titanosaurs were evidently established in western Africa (Mali) before the Cenomanian [238], and the only sauropods present in Cenomanian deposits in Egypt (*Aegyptosaurus*, *Paralititan*) are titanosaurs [239,240]. Finally, the Namba Member of the Galula Formation in Tanzania has produced the titanosaurs *Shingopana* and *Rukwatitan* [241,242]; however, the age of this stratigraphic unit is certain, with palaeomagnetic analysis indicating either a Cenomanian–Santonian or Campanian age [243].

*North America.* Despite their dominance in the Upper Jurassic Morrison Formation [244,245], neither camarasaurids nor diplodocoids are known from the Cretaceous of North America [246]. The only sauropod group that clearly straddles the Jurassic/Cretaceous boundary in North America is Brachiosauridae, with *Brachiosaurus* present in the Late Jurassic [247–250], and *Cedarosaurus* known from the Valanginian [251,252]. Otherwise, Berriasian–Valanginian sauropod faunas in North America are dominated by turiasaurians [253,254], a group for which there is currently no Jurassic—or post-Valanginian—record in North America. Barremian–lower Aptian deposits in Utah have produced the brachiosaurid *Venenosaurus* [255] and indeterminate titanosauriforms [256,257], and lower Aptian deposits in Maryland have produced abundant evidence of titanosauriforms [258–263]. The non-titanosaurian somphospondylan *Sauroposeidon* (=*Paluxysaurus*) spans the Aptian–Albian of Texas and Oklahoma [221,264–268], and possibly Arkansas [269]; other titanosauriforms recorded from this interval include indeterminate forms from Nevada, Montana and Wyoming [270–274], the brachiosaurid *Abydosaurus* [2] and the somphospondylan *Brontomerus* [64,275] from Utah, and a brachiosaurid possibly referable to *Cedarosaurus* [276–278], the somphospondylan *Astrophocaudia* [276,278] and indeterminate forms [276] from Texas. The geologically youngest pre-‘sauropod hiatus’ sauropod records in North America date to the late Albian–early Cenomanian [136,272]; these are the late-surviving brachiosaurid *Sonorasaurus* from New Mexico [279,280], and titanosauriform teeth from the Mussentuchit Member of the Cedar Mountain Formation of Utah [281].

*Europe.* Latest Jurassic sauropod faunas in western Europe were rather different from those of western North America. Both macronarians [64,282–284] and turiasaurians [285–290] were abundant and diverse, but diplodocoids were a minor component of the fauna, represented by a single diplodocid taxon [291,292] but no rebbachisaurids or dicraeosaurids. European earliest Cretaceous sauropod faunas appear to lack many of the forms that were present during the latest Jurassic, with no evidence for camarasaurid macronarians or flagellicaudatan diplodocoids. Berriasian deposits in the United Kingdom and Denmark have yielded sauropod teeth that are indeterminate, albeit not referable to either Diplodocoidea or Lithostrotia [293,294], whereas coeval deposits in France have produced indeterminate embryonic sauropod teeth [295] and remains of both turiasaurians and macronarians [296]. Slightly younger (upper Berriasian–lower Valanginian) deposits in the United Kingdom have yielded evidence of turiasaurians ([96]; although the exact provenance and age of this material is uncertain), the possible non-neosauropod eusauropod *Haestasaurus* [211,297–299], rebbachisaurids [300–302] and probable titanosauriforms [303–305]. Most Valanginian–Hauterivian sauropods from Europe (e.g. *Pelorosaurus*) are incomplete and difficult to classify [54,306–309]. However, there are exceptions in upper Hauterivian–lower Barremian deposits, including the titanosaur *Volgatitan* from western Russia [310], and the brachiosaurid *Soriatitan* from Spain [91]. Some Barremian-aged teeth from Spain are very similar to those of *Euhelopus* from Asia [82], possibly indicating a faunal link between the two continents at or before this time [311]. Other teeth from contemporaneous deposits in Spain represent macronarians, including somphospondylans [92,312–320]. The Barremian Wessex Formation of the United Kingdom has produced probable turiasaurians (possibly including *Oplosaurus armatus*) [6,305,321–323], rebbachisaurids [324,325], non-titanosaurian somphospondylans (e.g. *Ornithopsis*) and possibly brachiosaurids [326–331] and titanosaurs [64,66,305,332,333]. Collectively, this attests to high sauropod diversity in Europe during the Barremian. Upper Barremian–lower Aptian deposits in Spain have yielded rebbachisaurids [334,335] and titanosauriforms [336–339], including the probable somphospondylans *Tastavinsaurus* and *Europatitan* [340–342]. Upper Aptian deposits in the United Kingdom have yielded a possible turiasaurian tooth [343], which would be the stratigraphically youngest record of the group worldwide. Titanosaurs have been recognized in upper Aptian–lower Albian deposits in Italy [344], the Albian–Cenomanian of France (including *Normanniasaurus*; [345–347]), the middle–upper Cenomanian of Spain [339,348] and indeterminate macronarians (including *Macrurosaurus*) are known from the Albian–Cenomanian of the United Kingdom [349–351]. Very few sauropods (let alone dinosaurs) have been found in Turonian–Santonian deposits in Europe [136], although the presence of a relatively broad-crowned titanosauriform tooth in the Santonian of Hungary indicates that lithostrotians with narrow-crowned teeth might not have had the same stranglehold on Turonian–Santonian palaeoenvironments in Europe as they did in the rest of the world [352], a hypothesis further supported by the latest Cretaceous titanosaur *Ampelosaurus* from southwest Europe [353,354], which has anomalously relatively broad-crowned teeth [3].

*Asia.* Late Jurassic Asian sauropod faunas almost exclusively comprise non-neosauropodan forms, specifically mamenchisaurids [355,356], with one of the few possible exceptions being *Bellusaurus sui* [98,357], which might be an early-branching neosauropod [99,356]. The fact that Middle Jurassic deposits in China host the geologically oldest known neosauropod, the dicraeosaurid diplodocoid *Lingwulong shenqi*, implies either that neosauropods remained important faunal components in Asia throughout the Late Jurassic and that they have not been found owing to sampling biases, or that they diminished in importance in Asian faunas at this time [358].

Sauropods are well-represented in the earliest Cretaceous of Asia. Primary among these is *Euhelopus* from northeast China [67,68,82,359,360], which is Berriasian–Valanginian in age [361]. *Euhelopus* is generally regarded as a non-titanosaurian somphospondylan (e.g. [67]), but it has unusually broad teeth for a member of this clade and a recent study recovered it as a non-neosauropod eusauropod, with close affinities to the mamenchisaurids that dominated the Middle–Late Jurassic of China [362]. Berriasian deposits in Thailand have yielded *Euhelopus*-like teeth [73,363], as has the Berriasian–Barremian Oösh Formation of Mongolia [364]. Valanginian deposits in China have also produced teeth assigned to *Euhelopus* sp. [365]. If *Euhelopus* is (and teeth assigned to Euhelopodidae are) aligned with mamenchisaurids, then this would imply that Late Jurassic and earliest Cretaceous Asian sauropod faunas were dominated by that group; by contrast, if *Euhelopus* is an early-branching somphospondylan, then significant sauropod faunal turnover must have taken place. Irrespective of this, Valanginian-aged strata in Japan have yielded teeth superficially similar to those of *Euhelopus* that have been classified as Titanosauriformes indet. [1], as well as a partial somphospondylan skeleton [64,366,367].

Hauterivian–Barremian strata in South Korea have produced the probable somphospondylan ‘*Pukyongosaurus*’ [368,369], as well as a variety of broad-crowned teeth that pertain to titanosauriforms [1,370,371]. Similarly robust teeth have been reported from Barremian deposits in Japan [90,372] and Thailand [73,363], although the latter deposits have also yielded very narrow-crowned teeth [73,363,373,374], some or all of which might be referable to the non-titanosaurian somphospondylan *Phuwiangosaurus* [375–377]. The Barremian–lower Aptian Shengjinkou Formation of China has yielded three sauropod specimens interpreted to represent at least two separate taxa: *Silutitan* (a *Euhelopus*-like taxon), and the putative lithostrotian titanosaur *Hamititan* [378]. The upper Barremian–lower Aptian Yixian Formation of China has produced teeth assigned to cf. *Euhelopus* sp. [379], as well as somphospondylans [380], including *Liaoningtitan*, which has relatively narrow-crowned teeth [381]. By contrast, Barremian–Aptian deposits in Russia have produced the early diverging somphospondylan *Sibirotitan*, which has broad-crowned teeth [382], and the lithostrotian titanosaur *Tengrisaurus* [383,384].

The Barremian–Aptian Hekou Group of China hosts three titanosauriform taxa—*Huanghetitan* [385], *Daxiatitan* [386] and *Yongjinglong* [387]—only one of which (*Yongjinglong*) is represented by teeth (somewhat similar to those of *Euhelopus*). Several other sauropod teeth from the Barremian–Aptian of Asia, including some from China [388], and a ‘brachiosaurid’ from South Korea [389], are now regarded as indeterminate titanosauriforms [1], or, in the case of the ‘brachiosaurid’, as a euhelopodid [73]. By contrast, Aptian deposits in Thailand appear to preserve true titanosaurian teeth [73,363], and contemporaneous deposits in Laos have yielded the non-titanosaurian somphospondylan *Tangvayosaurus* [390]. Aptian deposits in Mongolia have produced indeterminate macronarian teeth [391] and other sauropod remains [392]. The sole sauropod tooth from the upper Aptian Sultanbobin Formation of Uzbekistan strongly resembles that assigned to cf. *Asiatosaurus mongoliensis* from Mongolia [391].

Chinese deposits of Aptian–Albian age have produced a plethora of sauropod fossils. The early-branching titanosauriform *Fusuisaurus* [393,394], the early-diverging somphospondylans *Qiaowanlong* [395,396] and *Liubangosaurus* [397], and indeterminate teeth assigned to ‘*Asiatosaurus*’ *kwangshiensis* [1,398], all hail from poorly constrained Aptian deposits, whereas upper Aptian deposits in China have yielded indeterminate sauropod remains [399] and *Mamenchisaurus anyuensis* [400]. The Aptian–Albian Shahai Formation in China has yielded teeth assigned to cf. *Euhelopus* [401,402], whereas the coeval On Gong Formation has produced the somphospondylan *Mongolosaurus* [69,72], which preserves both broad and narrow teeth. Four sauropod taxa have been established from the Aptian–Albian Haoling Formation: ‘*Huanghetitan*’ *ruyangensis* [403], *Ruyangosaurus* [404], *Xianshanosaurus* [405] and *Yunmenglong* [406]. Unfortunately, none of their holotypes preserve teeth. The first sauropod tooth reported from this stratigraphic unit, which is similar to those of brachiosaurids and *Euhelopus*, was tentatively assigned to *Xianshanosaurus* [405]; more recently, a fragmentary dentary bearing multiple teeth was described as being ‘phylogenetically intermediate’ between *Euhelopus* and *Liaoningotitan* [407].

Aptian–Albian deposits in South Korea have yielded only indeterminate sauropod remains [408–410]. By contrast, lower Albian deposits in Japan have produced the somphospondylan *Tambatitanis*, which preserves several fairly narrow-crowned teeth (average SI > 3.0) [90,411–413]. Lower Albian deposits from China have yielded similar isolated teeth [414], as well as the somphospondylan *Gobititan* [415]. The slightly younger (late Albian) Chinese titanosauriform *Borealosaurus* preserves a single tooth that is too worn for meaningful comparison [416]. The Albian–Cenomanian Longjing Formation has recently produced twisted teeth evincing the presence of a brachiosaurid, possibly implying mid-Cretaceous sauropod interchange between North America and Asia [417]. Many narrow-crowned teeth (SI often greater than 5.0) attributed to titanosaurs have been recovered in Uzbekistan from uppermost Albian/lower Cenomanian deposits, as well as the middle–upper Turonian Bissekty Formation [93,418,419]. A caudal vertebra from the Bissekty Formation, originally described as titanosaurian [419], was reinterpreted as rebbachisaurid and named *Dzharatitanis* [420], but even more recently transferred back to Titanosauria by Lerzo *et al*. [421]. However, the latter authors made no comparisons with contemporaneous Asian sauropods, such as *Dongyangosaurus* (see [131,422]), the caudal vertebrae of which show a striking similarity to that of *Dzharatitanis*, as originally noted by Sues *et al*. [419]. Turonian-aged strata in Japan have yielded indeterminate somphospondylan teeth that have intermediate SIs (ranging from 2.82 to 3.87) and possess high angle (approx. 70°) wear facets [423]. The precise age of the Chinese non-titanosaurian somphospondylan *Huabeisaurus* has proven difficult to determine, with estimates ranging from Cenomanian to Campanian [83,424]; given that it possesses fairly narrow-crowned teeth (SI approx. 3.4) that are somewhat similar to those of *Tambatitanis*, it is plausible that its true age is towards the stratigraphically older end of this range.

The presence of teeth similar to those of *Euhelopus* in multiple Lower Cretaceous deposits in China appears to support the notion of an effectively (but possibly not entirely [311]) endemic Asian clade of sauropods (Euhelopodidae) at this time, as proposed by D'Emic [66]. However, the constituents of this clade might differ markedly from that of Euhelopodidae *sensu* D'Emic [66]. If the recently revived hypothesis that *Euhelopus* is a late-surviving ‘mamenchisaurid’ (=euhelopodid) is correct [362], then both it and the *Euhelopus*-like teeth recovered from the Early Cretaceous of western Europe and Asia might simply indicate the survival of ‘Mamenchisauridae’ (=Euhelopodidae) in these regions well beyond the end of the Jurassic. Nevertheless, the removal of *Euhelopus* from this clade would not change the fact that Asia also hosted a unique group of early branching somphospondylan sauropods during the Early Cretaceous.

**Figure 10.**
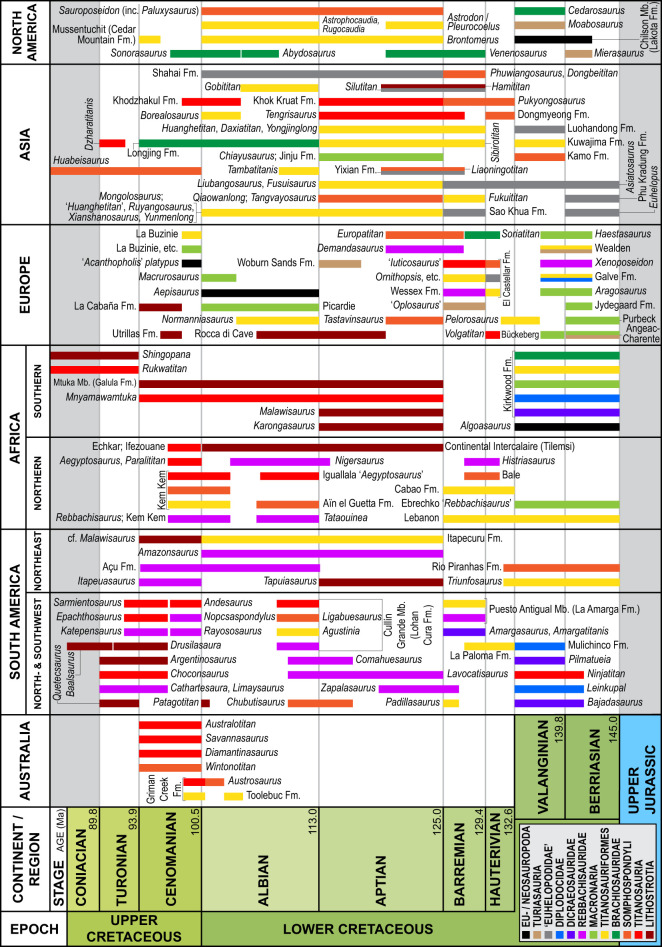
Chart of Berriasian–Turonian sauropod dinosaur spatio-temporal distribution. Note that the timespan indicated for each taxon does not necessarily represent its temporal distribution; more often than not, it simply indicates the range of possible ages that can be applied to the stratigraphic unit in which it was found. The Coniacian is only included on this chart so that uncertainty can be indicated in the stratigraphic ranges of *Baalsaurus*, *Huabeisaurus*, *Rukwatitan* and *Shingopana*. Sauropod taxa to which the oldest ascribed age is Coniacian have not been included.

*Summary:* The review above attempts to encapsulate the substantial sauropod turnover that took place during the Berriasian–Turonian on each continent. However, this information is perhaps best appreciated graphically (figure 10). Differentiation between the sauropod faunas of the various regions can be detected from the beginning of the Cretaceous. During the Berriasian–Valanginian interval, the following observations can be made: turiasaurians persisted in North America and Europe despite evidently going extinct elsewhere at the end of the Jurassic; somphospondylans made their first appearance in South America, including the earliest putative titanosaurs; flagellicaudatans (dicraeosaurids and diplodocids) were present in southern South America and southern Africa; macronarians (including titanosauriforms) prevailed in Europe alongside early rebbachisaurids; brachiosaurids persisted in at least North America and southern Africa; and ‘euhelopodids’ and somphospondylan titanosauriforms dominated Asian sauropod faunas at this time. The Hauterivian–Barremian is poorly represented in many regions. Despite this, Barremian deposits in southern South America preserve the geologically youngest dicraeosaurids known worldwide (and the oldest South American rebbachisaurids), whereas Eurasian strata from this stage host their oldest titanosaurs. The geologically youngest European brachiosaurids and rebbachisaurids date to the Hauterivian–Barremian and late Barremian–early Aptian respectively, and upper Aptian deposits in Europe host the youngest possible turiasaurian. Throughout the Hauterivian–Barremian, eastern Asian deposits are dominated by titanosauriforms—mostly early deriving somphospondylans. By the Aptian–Albian, faunal similarities between some regions, and differences between others, become increasingly evident: for example, rebbachisaurids flourished throughout southern South America and northern Africa, but whether or not they persisted in Europe is unclear. Brachiosaurids and ‘basal’ somphospondylans prevailed in North America to the exclusion of all other sauropods from the Aptian to the Cenomanian. Asia retained basal somphospondylans and titanosaurians in the Aptian–Albian, with brachiosaurids possibly present in the Albian–Cenomanian. And throughout Africa, northern South America and southern Europe, derived titanosaurs (=lithostrotians) started to proliferate from the Aptian. By the Cenomanian, the only sauropods left worldwide were rebbachisaurids (throughout South America and northern Africa), brachiosaurids (in North America and possibly Asia), early branching somphospondylans (in at least Australia, Asia and northern Africa) and titanosaurs (on all continents other than North America). Sauropods went extinct in North America at the end of the Cenomanian (until their reappearance during the Maastrichtian), whereas rebbachisaurids lingered in southern South America until their extinction at the end of the Turonian. From the Santonian–Maastrichtian, all sauropod remains known worldwide pertain to titanosaurs, and almost all of these pertain to Lithostrotia.

Within the Santonian–Maastrichtian interval, broad-crowned titanosaurian sauropod teeth are only known from Europe. Specifically, they have been reported from the Santonian of Hungary [352] and the Campanian–Maastrichtian of France and Spain [3,425,426]. The archipelagic nature of Europe throughout this interval might have enabled broad-toothed, early-deriving titanosaurs to persist alongside narrow-toothed forms, or perhaps emplaced a selective pressure upon some later-deriving titanosaurians that favoured the development of broader-crowned teeth.

## 6. Conclusion

The sauropod teeth recovered from the lower Upper Cretaceous Winton Formation to date do not conform to the morphology seen in later Cretaceous titanosaurs from South America and elsewhere, with the exception of some Santonian–Maastrichtian forms from western Europe. Given that members of Diamantinasauria display numerous postcranial characteristics that are considered plesiomorphic for Titanosauria (e.g. non-reniform sternal plates, manual phalanges, amphicoelous caudal vertebrae), this is perhaps not surprising. The referral of the teeth described herein to Diamantinasauria fortifies the interpretation that this clade occupies an early-branching position within Titanosauria. Although the sample size is currently quite small, the morphological homogeneity of the titanosaur teeth recovered from the Winton Formation suggests that sauropod ecomorphological diversity within this unit was relatively low—a range of tooth morphotypes equivalent to that seen in, for example, the Upper Jurassic Morrison and Tendaguru formations is not in evidence in the Winton Formation. Thus, the Winton Formation might plausibly document an exclusively titanosauriform sauropod fauna comprising non-titanosaurian somphospondylans and early-branching titanosaurs, or perhaps solely early-branching titanosaurs (diamantinasaurians).

## Data Availability

The electronic supplementary material, comprising 12 three-dimensional models of sauropod teeth, is available from Morphosource: https://www.morphosource.org/projects/000433637.
